# *N*,*N*-Diisopropyltryptamine and diptogenia: proposed mechanisms for a frequency-selective pitch-distorting psychedelic

**DOI:** 10.1093/braincomms/fcag093

**Published:** 2026-04-08

**Authors:** Aidan Hurst, Victoria M Bajo

**Affiliations:** Department of Physiology, Anatomy and Genetics, University of Oxford, Oxford OX1 3PT, UK; Department of Physiology, Anatomy and Genetics, University of Oxford, Oxford OX1 3PT, UK

## Abstract

*N*,*N*-Diisopropyltryptamine (DiPT) is a frequency-selective pitch-distorting psychedelic drug that induces a downward shift in perception where the auditory world of the subject sounds lower in pitch than in a sober state of consciousness. Hurst and Bajo propose the neural mechanisms for this effect and its use as potential therapeutic for tinnitus.

## Graphical Abstract

**Graphical Abstract:**
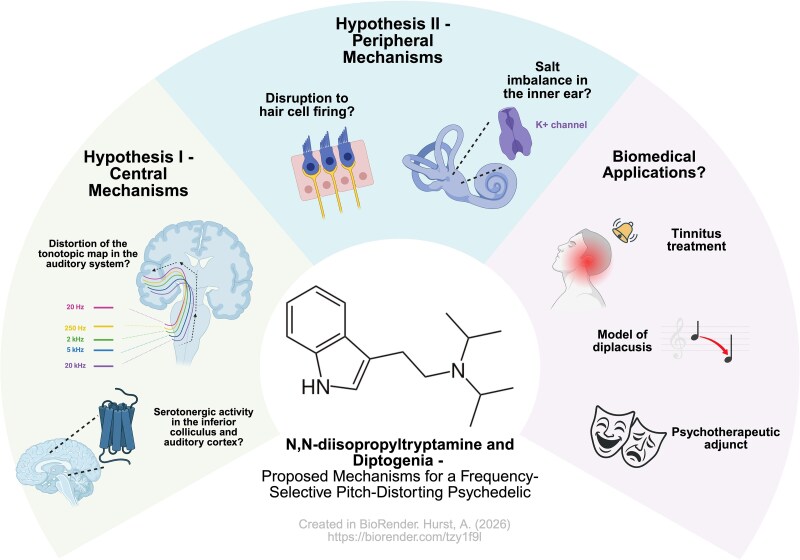

***The scientific study of the classical serotonergic psychedelics, like N,N-dimethyltrypamine (DMT), psilocybin, or lysergic acid diethylamide (LSD), has provided remarkable insights into the physiology of experience and emotion. Their ingestion induces radical changes to consciousness, affecting mood, personal insight, the sense of self, and the processing of sensory information, with many seeming to offer new avenues for the pharmacological alleviation of mental suffering. Amongst this class of compounds, the synthetic tryptamine* N,N*-diisopropyltryptamine (DiPT) stands as a remarkable exception. DiPT induces a unique downward shift in pitch perception, meaning the auditory world of the subject sound deeper (lower in pitch) than it is in a sober state of consciousness. This is an extraordinary state of consciousness not seen in any other drug nor psychopathology, and therefore, we coin this unique experiential effect ‘diptogenia’.***

Despite this unique effect, no published research to date has explored the mechanisms of its unique auditory distortion, providing a unique tool to illuminate insight into the mammalian auditory system. In this essay, we delineate a series of hypotheses for how *N*,*N*-diisopropyltryptamine (DiPT) may induce diptogenia in both the brain and periphery, and also discuss how DiPT may be harnessed as an experimental treatment for tinnitus, or as a pharmacological model of diplacusis.

## The phenomenological profile of DiPT

Our current understanding of DiPT’s subjective effects derive primarily from self-experiments, or *psychonautic bioassays,*^1^ collected from erowid.org—a repository containing a database of user-written reports written in non-laboratory, most often clandestine, settings. Whilst reports on this database involve considerable variability in dosage, compound purity, and social and psychological context, several consistent phenomenological features become apparent (see Erowid reference section for a short list of representative reports).

1. *Frequency-selective auditory effects:* DiPT produces a downward shift (diptogenia) that disproportionately affects *lower* frequencies whilst leaving higher frequencies intact. This frequency-dependent alteration suggests a mechanistic relationship to the tonotopic organization of the mammalian auditory system.
2. *Non-visual psychedelic effects:* Unlike the classical psychedelics, DiPT appears to lack psychedelic visual distortions like ‘wall breathing’, but seems to retain their emotionally clarifying effects, with some users describing it as ‘therapeutic’.
3. *Biphasic duration:* Emotional and cognitive effects persist for 4–8 h, which is consistent with other orally administered tryptamines, while diptogenia often continues for 24 h or more. The dissociation indicates that emotional clarity and diptogenia arise from physiologically distinct mechanisms.

These consistencies in DiPT’s phenomenology provide useful insights for foundational experiments in attempting to derive the compound’s mechanism of action.

## Hypothesis I: action upon the tonotopic map

To account for the frequency-specific nature of diptogenia, we first consider a central hypothesis—that DiPT’s frequency-selective pitch-distortion arises from its action across the entirety of the tonotopic map, reflecting the differential encoding strategies of high- and low-frequency sounds.

The function of the mammalian auditory system is to convert sound waves into a neural signal that gives rise to our perception of sound. In the inner ear’s cochlea, hair cells are arranged along a spiral basilar membrane that decode the frequency of the incoming sound wave into a neural signal. High-frequency sounds induce vibrations near the cochlea’s base, while low-frequency sounds vibrate towards the apex. Each hair cell is tuned to a specific range of frequencies. This forms the basis of the ‘tonotopic map’—where the physical location of cochlear vibration corresponds to the frequency of the incoming sound.^2^

**Figure 1:**
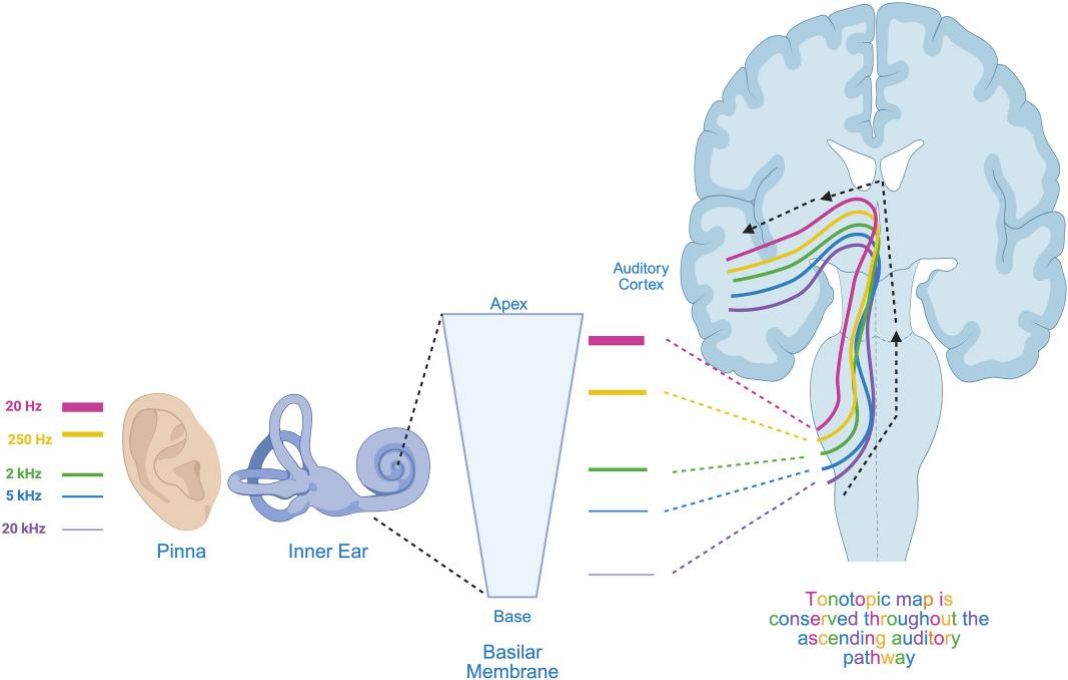
**Tonotopic map in the mammalian auditory system.** The tonotopic map is conserved throughout the ascending auditory system, and therefore, DiPT could have a broad inhibitory effect upon neural firing rates and induce a downward shift in frequency perception owing to the different coding styles of high and low frequency sounds. Hz, Hertz; kHz, kiloHertz. Created in BioRender. Hurst, A. (2026) https://BioRender.com/i9qw976.

The frequency-to-place organization of the cochlea is preserved throughout the entirety of the central auditory pathway. Auditory nerve fibres (ANFs) connect to hair cells, maintaining the cochlea’s tonotopic arrangement as neural signals travel from the inner ear to the brainstem, thalamus, and to the primary auditory cortex—this is known as the core or lemniscal auditory pathway (see Fig. 1; reviewed in^3^).

**Figure 2:**
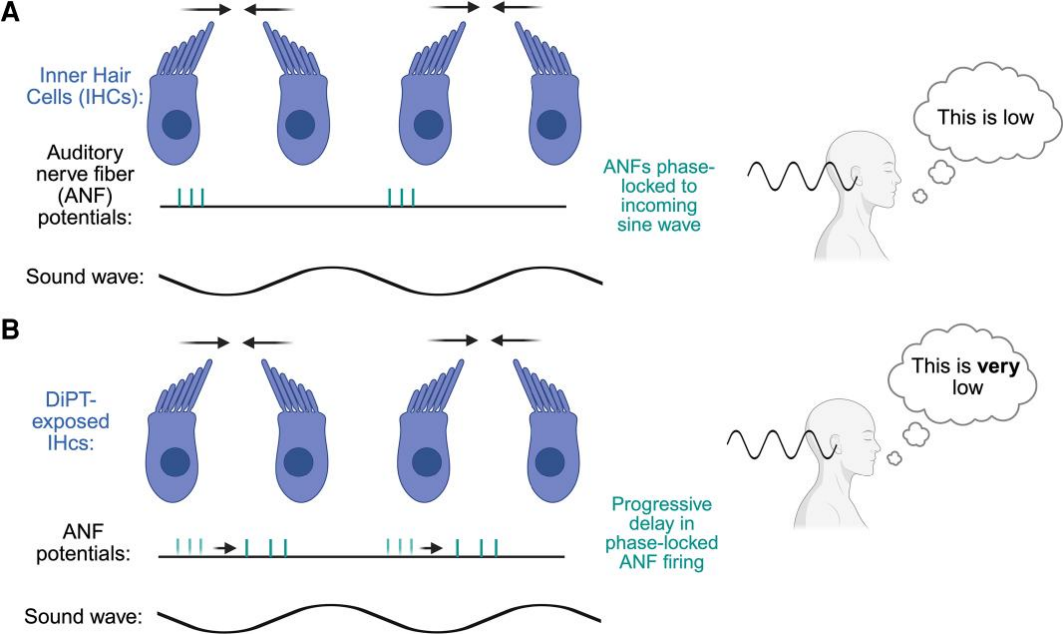
**Proposed effect of DiPT upon the firing rate of auditory nerve fibres (ANFs).** The perception of low-frequency sounds relies on phase-locking mechanisms in which auditory nerve fibres synchronize their firing to the sound wave’s frequency (panel A). DiPT may modulate this phase-locking process, by delaying the firing of ANFs with respect to the incoming sound wave. Under the influence of DiPT, the same sound frequency will sound deeper (panel B). Created in BioRender. Hurst, A. (2026) https://BioRender.com/4m3f866.

*The perception of low-frequency sounds depends upon a temporal coding mechanism:* ANFs synchronize their firing to match the rhythm of an incoming sound wave—a process called ‘phase-locking’,^4^ wherein the wafting of the stereocilia of inner hair cells (IHCs) synchronize to the phase of an incoming sound wave. We propose that DiPT may disrupt the timing synchronization in neurons tuned to lower frequencies. This could manifest in two ways: either neurons might fire less frequently during each cycle of the incoming sound wave, or their firing could be progressively delayed relative to the sound wave’s phase. Either alteration would make the brain interpret the sound as having a lower frequency than it actually does (Fig. 2).

*High-frequency sounds use a fundamentally different coding strategy:* High frequency sounds cannot be encoded through phase-locking since the refractory period of an action potential is too slow to match the exact frequency of the incoming sound wave.^5^ For high-frequency sounds, frequency is determined by which specific neurons are activated—this is known as *place coding.*^4^ If DiPT slows neural firing uniformly across the tonotopic map, high-frequency sounds would likely become quieter but retain their pitch, since their frequency information is carried by neuronal identity rather than firing timing, whilst their loudness is encoded via a rate code. The underlying mechanism of these changes may involve DiPT's modulation of uncharacterized receptors or ion channels that are expressed across the tonotopic map.

Tonotopy is not only established in the varying width and thickness of the basilar membrane and the gradual volume of perilymph moved by the vibration at the oval window, but also reinforced through gradients in the IHC ribbon synapse size, the number of active zones per IHC, and in differential expression of ion channels across the cochleotopic axis. IHC neurotransmission primarily depends upon L-type calcium channels, like Cav1.3, which are expressed presynaptically and enable rapid glutamate release at the ribbon synapse—one of the fastest and most rapidly recycling synapses in the vertebrate nervous system.

DiPT may act upon an IHC-specific G protein-coupled receptor (GPCR)—of which more than thirty are expressed in the murine IHC GPCR-tree mapper (GPCRome)—whose downstream signalling cascade indirectly inhibits Cav1.3. This interference would disrupt the synchrony between stereocilia-driven depolarization and subsequent glutamate release onto ANFs.

Furthermore, each IHC contains 5–30 active zones depending on species and its respective location along the tonotopic axis, with their density peaking at the tonotopic region with the greatest sensitivity to sound. These specialized synapses require rapid vesicle recycling in order to sustain firing rates at hundreds of Hertz. If DiPT reduces calcium influx through presynaptic channels or modulates a receptor that inhibits them, this could slow IHC firing and produce the tonotopic distortion characteristic of diptogenia. Consequently, the fidelity of phase-locked action potentials would diminish, leading to tones perceived as lower in frequency and disrupting synchrony between ANF firing and the incoming sound wave.

More centrally, DiPT’s serotonergic action might affect tonotopic encoding in the inferior colliculus, as it has been observed that serotonin 1A (5-HT1a) receptor agonists 8-hydroxy-2-(di-*N*-propylamino)-tetralin (8-OH-DPAT) can selectively depress auditory-evoked responses, with a greater effect on neurons exhibiting longer first-spike latencies and on later spikes in a spike train. It should be noted that other psychedelic 5-HT1a agonists have not been reported to cause diptogenia, but DiPT’s serotonergic action may simply confound its auditory effects whose *locus* is in ANFs or the inner ear.

## Hypothesis II: peripheral action and pharmacokinetic considerations

We also hypothesize that DiPT’s biphasic experiential profile—where emotional effects last 6–8 h while diptogenia persists for more than 24 h—might reflect differential drug distribution and clearance between the central nervous system (CNS) and inner ear. The emotional effects likely stem from its serotonergic and monoaminergic action in the CNS, whilst the prolonged pitch-distortion could reflect long-lived peripheral alterations to cochlear biomechanics, disruptions to endocochlear potential (EP) homeostasis, or the synaptic timing of IHCs.

The 6–8 h duration of DiPT’s emotional effects aligns with other structurally similar tryptamines like diethyltryptamine (DET), which are metabolized via cytochrome P450 2D6 (CYP2D)-mediated hydroxylation.^6^ DiPT likely follows a similar metabolic pathway, allowing relatively rapid clearance from the CNS. In contrast, diptogenia may result from DiPT's accumulation in the inner ear, where drug clearance occurs much more slowly due to its complex anatomy and limited vascularization where drugs are retained longer than in vascularly active brain regions.^7^ DiPT's lipophilic properties should facilitate passage across the blood-labyrinth barrier (BLB), allowing access to cochlear structures, the basilar membrane, perilymph or endolymph.

The emotionally cathartic nature of DiPT's emotional effects can be explained by its broad neuropharmacological profile. All studies to date have shown that DiPT binds with negligible affinity to the 5-HT2a receptor, which is the key receptor that mediates the ‘classical psychedelic syndrome’. This might explain why DiPT lacks a significant visual component, and why it induces significantly fewer head-twitch responses when compared to its classically psychedelic isomer *N*,*N*-dipropyltryptamine (DPT).^8^ The nature of the emotional and cognitive effects likely arises from DiPT's activity at CNS receptors, including 5-HT1a, 5-HT2b, 5-HT7, imidazoline, and dopamine D3 receptors,^9^ which mediate psychedelic-like emotional states. It also inhibits serotonin and vesicular monoamine transporter 2, without appreciable interaction at dopamine or noradrenaline transporters at concentrations below 10 µM.^10^

Diptogenia resembles the pitch shift seen in the aural disease diplacusis binauralis, whereby a tone is perceived as different pitches between ears. Like DiPT, diplacusis rarely affects the entire tonal scale to an equal degree, and is hypothesized to be caused by a segment of the membrane which normally vibrates to a certain tone to vibrate to a tone of a higher or lower pitch. With this phenomenological similarity, it connotes that diptogenia may be of fundamentally mechanical, and not purely neurological, origin—for example, in the alteration of cochlear stiffness. It would be worth assessing whether DiPT could serve as a pharmacological model of diplacusis.

DiPT could feasibly disrupt ion transport within the stria vascularis—the cochlear structure responsible for generating the EP and the high potassium concentration in the endolymph. This could occur through disruption either direct or indirect (via a GPCR second messenger) disruption to potassium voltage-gated channel subfamily KQT member 1/E regulatory subunit 1 (KCNQ1/KCNE1) channels, Kir4.1 potassium inward rectifiers, and sodium-potassium adenosine triphosphatase (Na^+^,K^+^-ATPase) pumps, or sodium-potassium-chloride (NKCC1) cotransporters, all of which are involved in the recycling of potassium from perilymph to endolymph. By collapsing the ionic gradient that these channels establish, the EP will reduce, disrupt the synchrony of IHC firing with the phase of an incoming sound wave, and the sensitivity of the IHC’s wafting of the stereocilia. This ionic gradient collapse could also affect the hydraulics of the endolymph and perilymph and impede basilar membrane vibration.

## Potential biomedical applications and neuroscientific implications

DiPT's unique ability to selectively lower pitch perception could offer a novel therapeutic approach to tinnitus, where patients experience persistent high-frequency phantom sounds. Tinnitus disproportionately affects war veterans exposed to explosive blasts and germ line cancer patients undergoing platinating chemotherapies. The origin of tinnitus in these cases is often associated with psychological trauma or existential fear, like post-traumatic stress disorder or end of life anxiety, which psychedelic therapies show tremendous promise in relieving. It may make sense, therefore, that DiPT could alleviate the ringing percept of tinnitus and its associated mental suffering. Rather than masking tinnitus’ ringing hallucination, DiPT could potentially normalize it by inducing a compensatory downward frequency shift. Emotional integration of the psychological trauma that caused the tinnitus could be pharmacologically facilitated by DiPT’s serotonergic psychedelic activity.

DiPT’s use as an adjunct to psychotherapy may be hindered in nations where tonal languages are spoken, as tonal languages convey word meaning through pitch—the sensory domain that DiPT selectively distorts. This is an especially important consideration given that 60–70% of human languages are tonal.

Could understanding DiPT’s mechanism lead to developing more selective compounds that target specific frequency ranges without the broader psychoactive effects? And if so, what strategies would medicinal chemists use to disentangle diptogenia from its psychoactivity? Can DiPT expand the perceptual boundaries of pitch, and create the subjective experience of tones that are deeper than the lowest sober percept, effectively generating the subjective experience of ‘deeper than the deepest’ tones?

Beyond therapeutic applications, diptogenia may enable the design of novel musical instruments that emit sounds tailored to diptogenic perception, or in the invention of cryptic tonal languages that exploit diptogenia as part of their communication strategy. A detailed understanding of DiPT’s mechanism of action will serve to correlate biochemistry with phenomenology, enabling us to understand how the biochemical architecture of the brain and body serves as a substrate for our experience of the inner world.

## Data Availability

No new data were generated or analysed in support of this research.
